# Variant antigen repertoires in *Trypanosoma congolense* populations and experimental infections can be profiled from deep sequence data using universal protein motifs

**DOI:** 10.1101/gr.234146.118

**Published:** 2018-09

**Authors:** Sara Silva Pereira, Aitor Casas-Sánchez, Lee R. Haines, Moses Ogugo, Kihara Absolomon, Mandy Sanders, Steve Kemp, Álvaro Acosta-Serrano, Harry Noyes, Matthew Berriman, Andrew P. Jackson

**Affiliations:** 1 Department of Infection Biology, Institute of Infection and Global Health, University of Liverpool, Liverpool L3 5RF, United Kingdom;; 2 Department of Parasitology, Liverpool School of Tropical Medicine, Liverpool L3 5QA, United Kingdom;; 3 Department of Vector Biology, Liverpool School of Tropical Medicine, Liverpool L3 5QA, United Kingdom;; 4 International Livestock Research Institute, Nairobi 00100, Kenya;; 5 Wellcome Trust Sanger Institute, Wellcome Genome Campus, Hinxton CB10 1SA, United Kingdom;; 6 Institute of Integrative Biology, University of Liverpool, Liverpool L69 7ZB, United Kingdom

## Abstract

African trypanosomes are vector-borne hemoparasites of humans and animals. In the mammal, parasites evade the immune response through antigenic variation. Periodic switching of the variant surface glycoprotein (VSG) coat covering their cell surface allows sequential expansion of serologically distinct parasite clones. Trypanosome genomes contain many hundreds of *VSG* genes, subject to rapid changes in nucleotide sequence, copy number, and chromosomal position. Thus, analyzing, or even quantifying, VSG diversity over space and time presents an enormous challenge to conventional techniques. Indeed, previous population genomic studies have overlooked this vital aspect of pathogen biology for lack of analytical tools. Here we present a method for analyzing population-scale VSG diversity in *Trypanosoma congolense* from deep sequencing data. Previously, we suggested that *T. congolense* VSGs segregate into defined “phylotypes” that do not recombine. In our data set comprising 41 *T. congolense* genome sequences from across Africa, these phylotypes are universal and exhaustive. Screening sequence contigs with diagnostic protein motifs accurately quantifies relative phylotype frequencies, providing a metric of VSG diversity, called the “variant antigen profile.” We applied our metric to VSG expression in the tsetse fly, showing that certain, rare VSG phylotypes may be preferentially expressed in infective, metacyclic-stage parasites. Hence, variant antigen profiling accurately and rapidly determines the *T. congolense VSG* gene and transcript repertoire from sequence data, without need for manual curation or highly contiguous sequences. It offers a tractable approach to measuring VSG diversity across strains and during infections, which is imperative to understanding the host–parasite interaction at population and individual scales.

Many blood-borne pathogens survive in mammalian hosts through antigenic variation, i.e., the sequential replacement of their surface proteins to render antibody responses redundant (Namangala 2011; Matthews et al. 2015). This type of immune evasion typically requires a reservoir of diversity in the form of alternative variant antigens. Genome sequencing has revealed the complexity of variant antigen gene families, e.g., *var* genes encoding the PfEMP1 proteins of *Plasmodium falciparum* (Baruch et al. 1995; Smith et al. 1995; Su et al. 1995) and *vls* genes in *Borrelia* spp. (Norris 2014). Analysis of these genes on genomic and population scales is a challenge. Here, we present a solution for characterizing variant surface glycoprotein (VSG) repertoire in *Trypanosoma congolense*.

African trypanosomes are extracellular hemoparasites of humans and animals that are transmitted by blood-feeding tsetse flies (*Glossina* spp.). Animal African trypanosomiasis (AAT) is a common, endemic disease across sub-Saharan Africa and an expanding, epidemic disease in South America (Morrison et al. 2016). AAT causes a potentially lethal syndrome of inflammatory anemia and neurological dysfunction and is responsible for severe mortality and morbidity among African livestock, as well as considerable economic loss to many developing economies. *T. congolense* (Savannah subtype) is the most prevalent and pathogenic species in African livestock, with an extensive host range (van den Bossche et al. 2011). At present, disease control options are inadequate due to trypanocidal drug resistance (Melaku and Birasa 2013) and antigenic variation that precludes vaccine development.

The *T. congolense* lifecycle begins with a tsetse fly feeding on an infected vertebrate host. The ingested parasite differentiates into a procyclic form in the fly midgut and, after developing in the proventriculus, migrates to the mouthparts. Here, it develops first into an epimastigote stage and then into an infective metacyclic form. The metacyclic parasites are transmitted to another animal via the saliva when the fly feeds again. Metacyclic and bloodstream-stage trypanosomes are coated by a VSG monolayer, which conceals most invariant surface molecules from the immune system (for review, see Schwede et al. 2015). Strong humoral responses are mounted against each VSG, but these are not typically protective against other parasite strains expressing alternative variant antigens (Nantulya et al. 1980; Akol and Murray 1983; Frame et al. 1990). This causes antigenic variation, in which high-abundance parasite clones are replaced within the infrapopulation by other, low-abundance clones that express antigenically distinct VSG (for reviews, see Horn 2014; Matthews et al. 2015). Antigenic variation prevents any long-term protective immunity and results in a chronic infection. Hence, long-term persistence of the parasite population requires a ready supply of antigenic diversity.

The dynamics of VSG expression have been documented using transcriptomic profiling of murine infections by *Trypanosoma brucei* laboratory strains. These experiments indicated that these dynamics are more complex than previously thought, due to early appearance of novel sequence variants (Hall et al. 2013) and the multiplicity of dominant clones within the same parasite infrapopulation (Mugnier et al. 2015). These studies show the value of understanding the expression of large VSG repertoires, the dynamics of which are crucial to understanding antigenic variation itself. However, characterization of hundreds of VSG is a daunting process and has so far only been undertaken for a few genomes (Marcello and Barry 2007; Jackson et al. 2010, 2012; Hall et al. 2013; Cross et al. 2014; Mugnier et al. 2015) and never beyond the context of a single strain. Recent population genomics studies of African trypanosomes, i.e., *T. brucei* (Sistrom et al. 2014; Weir et al. 2016) and *T. congolense* (Tihon et al. 2017), have overlooked VSG diversity, perhaps because existing read-mapping methods cannot be safely applied to labile VSG loci. Yet, besides antigenic variation, VSG and related genes are crucial to parasite host range and virulence (Pays 2006). VSG are implicated in resistance to complement-mediated lysis (Ferrante and Allison 1983; Devine et al. 1986), antibody clearance (Engstler et al. 2007), and cytokine dysregulation among innate immune cells, which ultimately leads to immune suppression and disease symptoms (Vincendeau and Bouteille 2006). Hence, the molecular dynamics of antigenic variation during infections, as well as the functional differentiation among VSG, are central to understanding AAT; to examine these, we need an automated method for VSG identification from systems data, integrating a reliable systematics.

Similar initiatives were critical to understanding transmission and clinical phenotypes of other antigenically variable organisms. For instance, antigen mapping of hemagglutinin has improved the prediction of antigenic drift in influenza A (McHardy and Adams 2009), which is vital for the success of the influenza vaccine. Likewise, a precise understanding of the genetic diversity of HIV envelope glycoproteins preceded the formulation of a multivalent subunit vaccine (McCutchan et al. 1996). In malaria, molecular epidemiological studies of *var* gene diversity in *P. falciparum* have uncovered strong links between particular *var* genes, infection reservoirs, and disease severity (Chen et al. 2011; Wang et al. 2012).

In this study, we aimed to develop an approach for analyzing *Trypanosoma congolense* VSG diversity in deep sequencing data, producing what we call a “variant antigen profile” (VAP). Previously, we showed that *T. congolense* VSG sort into 15 clades, many of which predate the species’ origin and between which no recombination is evident (Jackson et al. 2012). We first confirm that these clades are a feature of all strains and then exploit their regularity to examine variation in VSG repertoire across *T. congolense* clinical isolates and during experimental infections.

## Results

### The *T. congolense* VSG repertoire consists of 15 phylotypes

We have sequenced the genomes of 41 T*. congolense* clinical isolates from different animal species originating from six African countries over a 31-yr period (1961–1992) (Supplemental Table S1). *VSG* genes were identified in the assembled genomes by sequence homology with typical a-type VSG (i.e., *T. brucei* a-type VSG and the *T. congolense* IL3000 transferrin receptors) and b-type VSG (i.e., *T. congolense* IL3000 VSG and *T. brucei* ESAG2) (Carrington et al. 1991; Marcello and Barry 2007; Jackson et al. 2012). A significance threshold of *E* < 0.001, sequence similarity ≥40%, and length 150 or more amino acids was applied to remove low-confidence matches; all retrieved sequences were manually curated. Only b-type VSGs were found, confirming that *T. congolense* lacks a-type VSGs (Jackson et al. 2012). VSG sequences from each strain were aligned with the reference (IL3000) VSG repertoire. Previously, we have divided *T. congolense* IL3000 *VSG* genes into 15 distinct phylotypes (Jackson et al. 2012); these are denoted by “*P*” hereafter. Phylogenies were estimated from VSG alignments of each strain, and these indicated that all strain VSGs clustered robustly within the established clades. No novel phylotypes were found, and VSGs from all *T. congolense* strains may be accommodated by the established cladistic typology.

**Figure 1.**
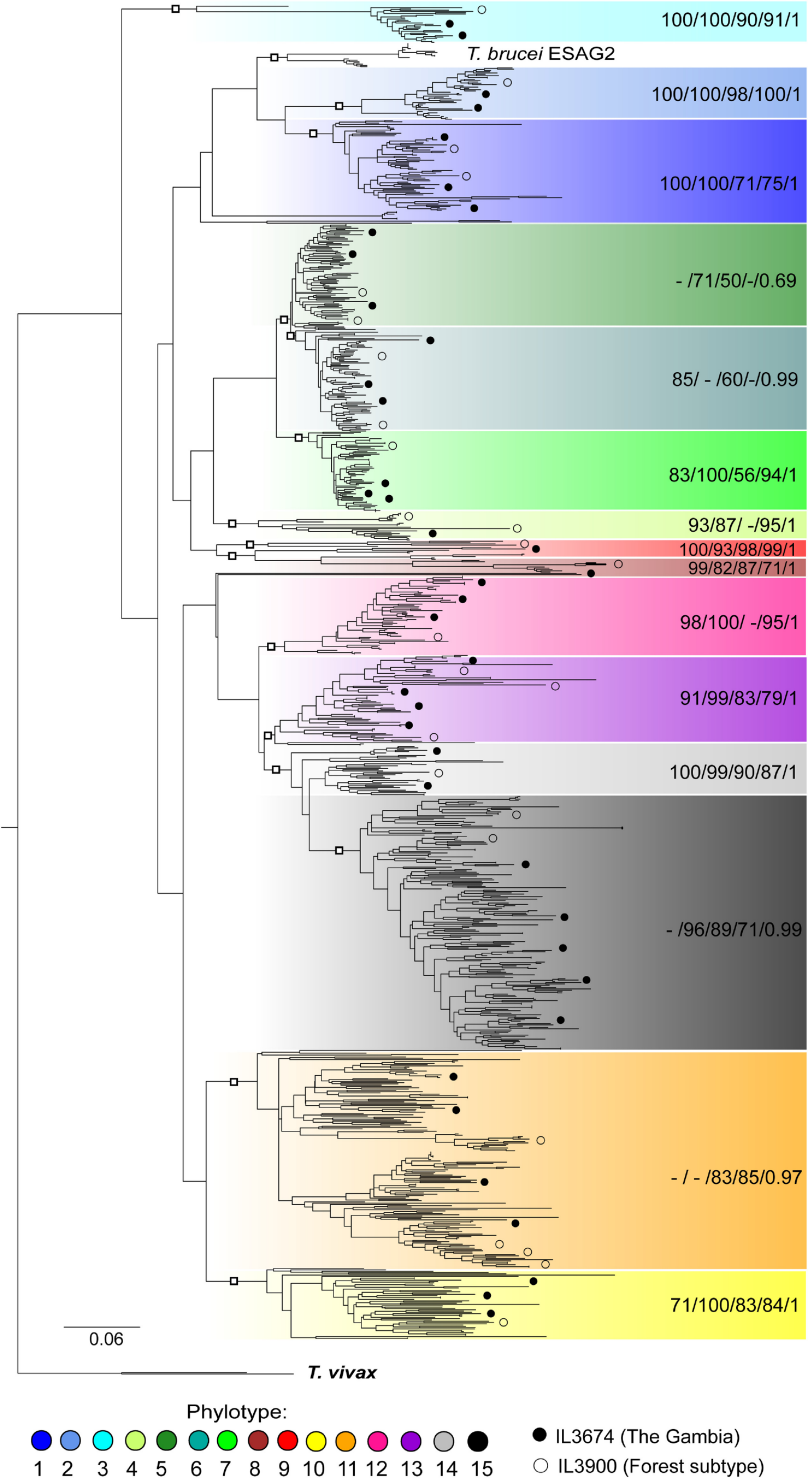
Maximum likelihood (ML) phylogeny of *T. congolense* VSGs. The phylogeny was estimated from full-length VSG protein sequences of IL3000 (Kenya), IL3674 (The Gambia), and IL3900 (Forest subtype, Burkina Faso) with RAxML (Stamatakis 2014) using a ML method with a WAG+Γ model and 100 bootstrap replicates. The 15 phylotypes identified in IL3000 are color-coded according to the key. Positions of example sequences from IL3674 and IL3900 are indicated according to the key. Labels for the internal nodes of each phylotype (marked by the open squares) are shown on the *right*. These labels indicate the bootstrap percentages for ML from the complete tree (RAxML), as well as ML (PhyML) (Guindon et al. 2010), ML (MEGA7) (Kumar et al. 2016), neighbor joining (NJ) (Felsenstein 1989), and posterior probabilities (BI) (Huelsenbeck and Ronquist 2001) estimated from a pruned tree containing 147 sequences. Tree is rooted with two *T. vivax* VSG sequences (Fam23).

To demonstrate, we re-estimated the *T. congolense* VSG phylogeny by combining the VSG repertoires of one West African strain (IL3674), one *T. congolense* Forest-type (IL3900), and the reference strain (IL3000) (Fig. 1; Gibson 2012). These strains were chosen because they encompass the greatest geographical and genetic distances in our sample set. To confirm that strain VSG sequences could be robustly placed within established phylotypes, we conducted log-likelihood ratio tests on their positions. For a given VSG, the log-likelihood of an unconstrained tree was compared with another in which the VSG was constrained within the sister clade of the observed position. Log-likelihood ratio tests were conducted on all VSG sequences from both the IL3674 and IL3900 strains, in triplicate for each clade. Negative log-likelihood of unconstrained trees was significantly higher than constrained trees in all cases (*P* < 0.01), except for the IL3900 *P2*. This was significantly different from the adjacent *P1*, though not from the adjacent *P3*. These tests confirm that the positions of strain VSG within the 15 VSG phylotypes seen in *T. congolense* IL3000 are robust.

Thus, within our diverse sample set, the 15 established phylotypes are both universal and exhaustive. These results suggest that these phylotypes can be used to describe the VSG repertoire of any *T. congolense* strain.

### The VAP describes VSG repertoires from deep sequencing data using protein motifs

**Figure 2.**
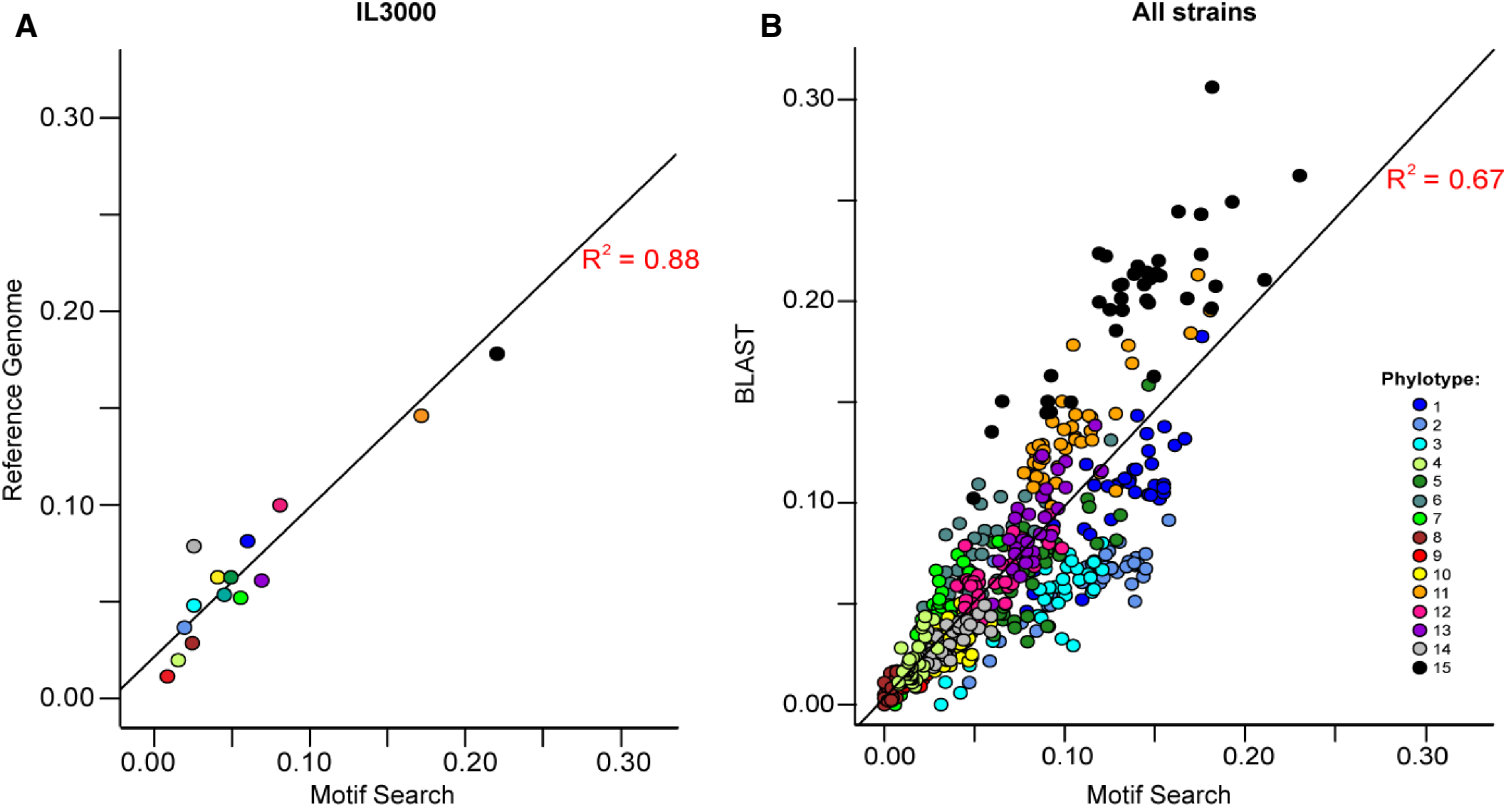
Performance of the protein motif-based variant antigen profile (VAP). (*A*) Correlation of motif-based and manually curated phylotype frequencies in the *T. congolense* IL3000 reference genome sequence. Pearson's product moment correlation statistics: *R*^2^= 0.88, *t*_(13)_ = 9.7321, *P* < 0.001. (*B*) Correlation of motif-based and manually curated phylotype frequencies in 41 T*. congolense* strains. Manual VAPs were estimated by counting the top matches from BLASTx (Altschul et al. 1990). Pearson's product moment correlation: *R*^2^= 0.64, *t*_(566)_ = 34.39, *P* < 0.001. Phylotypes are color-coded according to the key.

We adopted, as universal features of the *T. congolense* VSG repertoire, the 15 phylotypes as the basis of “variant antigen profiling” (VAP), a metric of VSG diversity based on the relative frequencies of each phylotype in a genome or transcriptome. Typically, the VSG repertoire is characterized manually using sequence similarity searches. However, this is time-consuming and requires technical expertise to detect sequence identification errors inherent to divergent gene families. To make this task simpler, we focused on protein motifs unique to each phylotype. We have identified 28 diagnostic motifs of nine to 79 amino acids (Supplemental Fig. S1). These were identified heuristically and evaluated by their ability to recover the observed VSG phylotype frequencies in the *T. congolense* IL3000 reference genome sequence. The C-terminal domains of *T. congolense* VSG are less variable than the N-terminal domains; therefore, most of the motifs (20/28) were selected from the C-terminal domains. However, as there is no recombination between phylotypes that would exchange N termini (Jackson et al. 2012), the latter are coupled with the C-terminal motifs, which therefore produce an accurate profile of the whole molecule. The protein motifs were described in hidden Markov models (HMMs) and used to screen six-way translations of sequencing data with HMMER3 (Eddy 2009). Phylotype frequencies inferred by the final motifs correlate well to the manually curated IL3000 repertoire (*R*^2^ = 0.88, Pearson's product moment correlation, *t*_(13)_ = 9.7321, *P* < 0.001) (Fig. 2A).

We have evaluated motif performance by comparing manually annotated antigen profiles (tBLASTx) to profiles produced with the novel HMMs for the 41 isolates. Although we observed a good correlation between the methods (*R*^2^ = 0.67, Pearson's product moment correlation, *t*_(566)_ = 34.4, *P* < 0.001) (Fig. 2B), there were clear differences in phylotype frequency. Disagreement between BLAST-based and motif-based profiling occurs because of differences in how the methods treat both large contigs containing multiple VSG and small VSG fragments resulting from poor assembly. In the first case, BLAST allocates contigs to phylotypes based on the VSGs with the highest similarity value, whereas the motif search allocates all VSGs on the contig according to the presence of structural motifs. This meant that the motif searching method recovered more VSGs than the sequence similarity method (mean ± σ = 721 ± 277 vs. 669 ± 292, paired *t*-test, *P*-value = 0.005). In the second case, of genomes with poorer assemblies, (i.e., greater VSG fragmentation), BLAST allocates each VSG fragment to a phylotype even when fragments belong to the same gene, which skews the profile when the fragmentation level is not the same for all phylotypes. In fact, our motif-searching method robustly returns a known VAP when VSG sequences are fragmented to ≥40% of the original gene length (223 nucleotides) (see Supplemental Methods). It is also robust in situations of partial genome coverage, estimating an accurate VAP for *T. congolense* IL3000 even with only 30% of known VSGs (see Supplemental Methods).

### The genomic VAP is a stable but variable measure across the population

**Figure 3.**
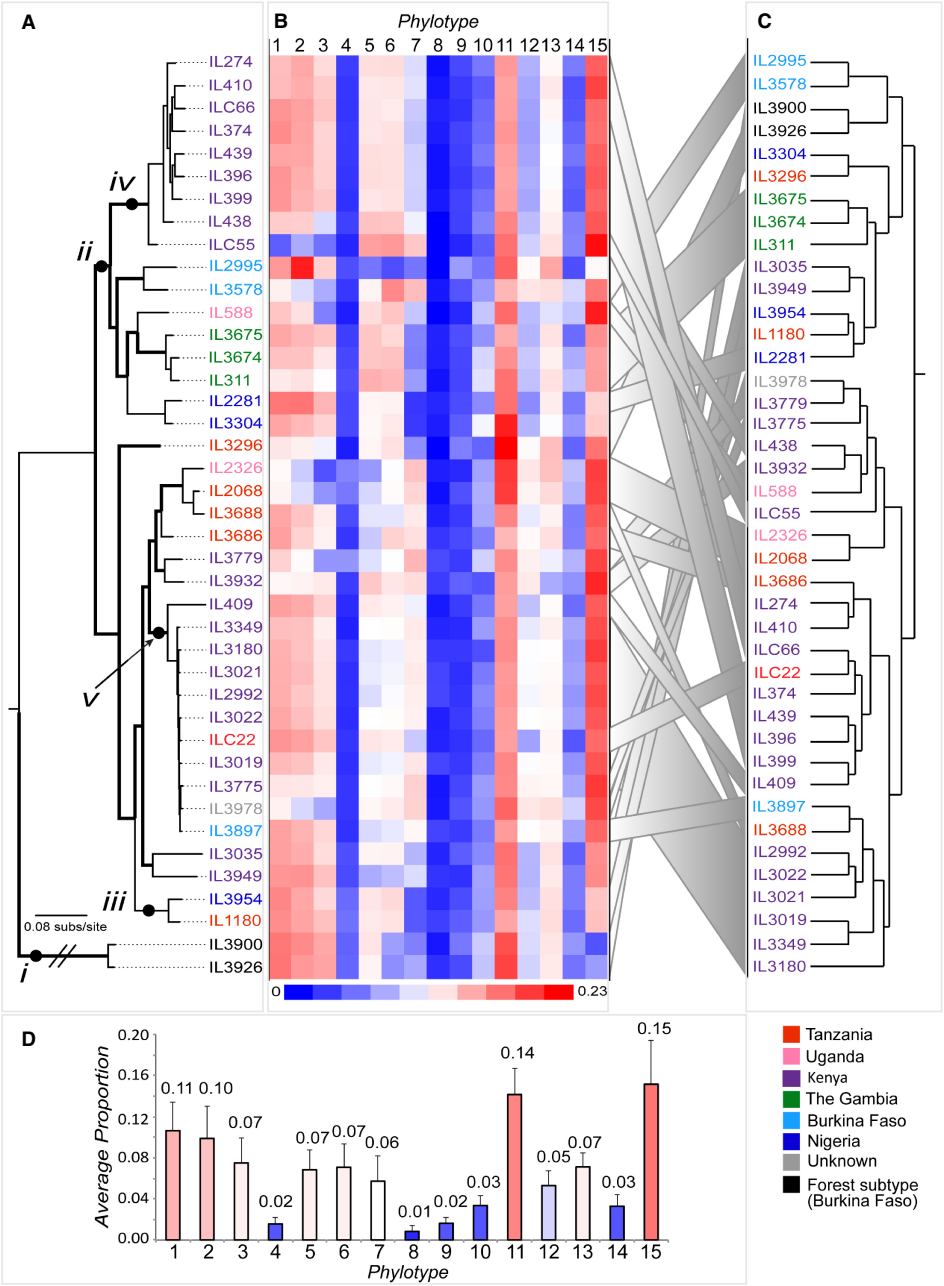
Relationships between the VSG repertoire, geography, and population structure in *T. congolense*. (*A*) ML phylogeny of *T. congolense strains* in this study based on whole-genome single-nucleotide polymorphisms (SNPs), estimated with RAxML (Stamatakis 2014) with a GTR + Γ model and 100 bootstrap replicates (branches with bootstrap greater than 70 are shown in bold). Labels “*i*” to “*v*” denote examples referred to in the text. Label “*i*” shows the long phylogenetic distance between *T. congolense* Savannah and Forest subtypes; “*ii*” points to the only clade maintaining a geographic signature. Labels “*iii*,” “*iv*,” and “*v*” show examples of lack of concordance between the population history recapitulated by the SNP phylogeny and the VAP, demonstrated by the dendrogram. (*B*) VAPs for all strains shown as a heatmap of the proportions of 15 universal phylotypes. (*C*) A dendrogram depicting the relationships among VAPs based on Euclidian distances estimated in R. Gray ribbons link the position of parasite strains in *A* and *C*. (*D*) A bar chart showing the average proportion of each phylotype (mean ± σ) across all strains. Strains are color-coded by provenance according to the key.

The global scale of *VSG* gene diversity is commonly thought to be large; in comparable systems, such as the *P. falciparum var* genes, populations can mutually exclusive repertoires of variant antigens (Chen et al. 2011). To examine this issue, we estimated VAPs for each genome in our data set. Our results show that the composition of the VAP is stable across *T. congolense* isolates (Fig. 3B). Particular phylotypes are consistently the most numerous in the genome (i.e., *P1*, *P11*, and *P15*), while others are consistently scarce (i.e., *P4*, *P8*, and *P9*) (Fig. 3D). To assess whether the stability observed was statistically significant, the observed frequencies were compared to 41 simulated VAPs, each estimated from 250 VSGs randomly selected from all strain VSGs. Simulated VAPs showed significantly more variation than observed VAPs (*F*-test, *P* < 0.001) (Supplemental Fig. S2), indicating that VSG repertoire is not subject to random drift across the population.

**Figure 4.**
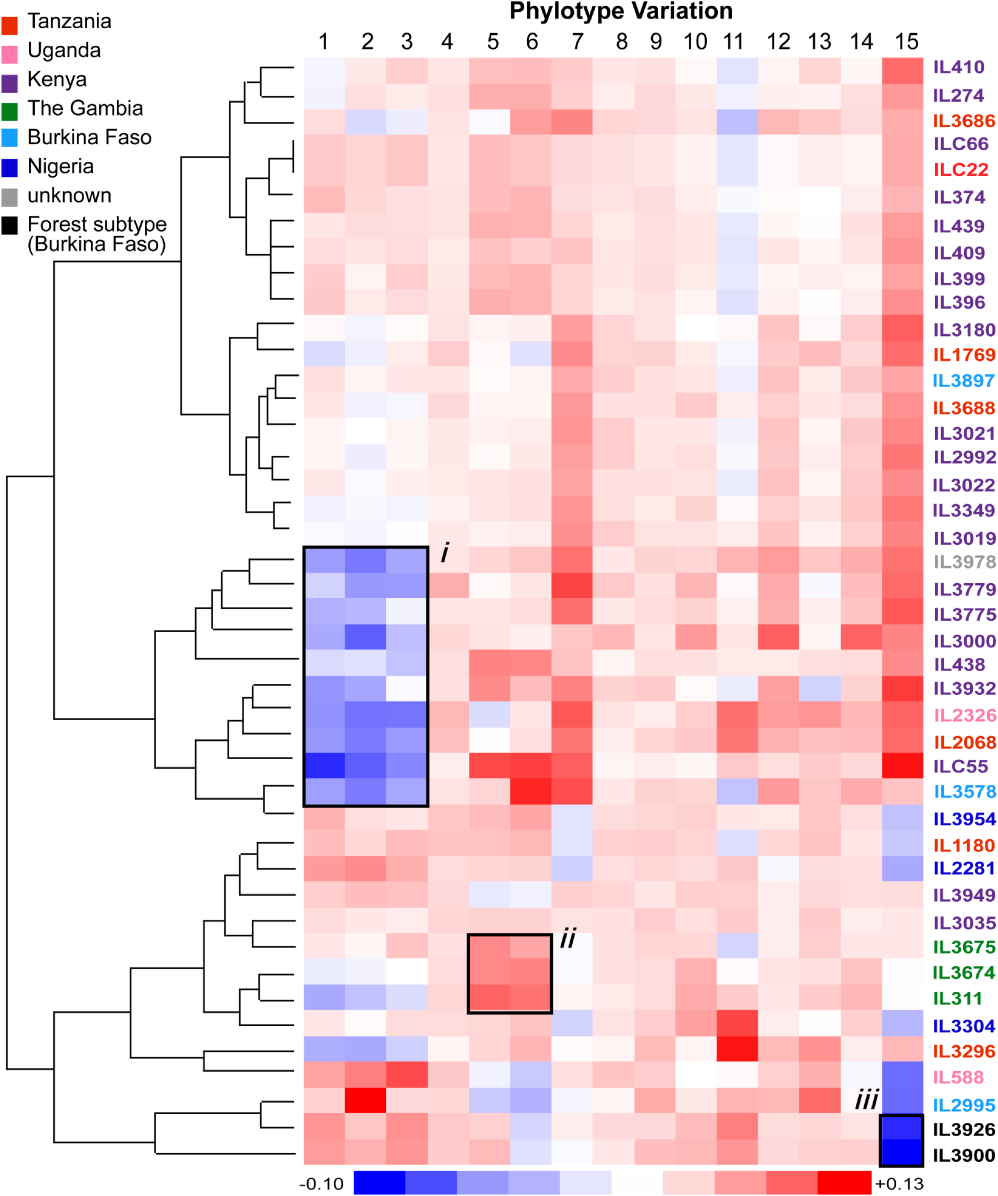
Phylotype variation across the sample cohort. The heatmap represents phylotype variation across the sample cohort expressed as the deviation from the mean. The dendrogram reflects the relationships among the VSG repertoires of each strain. Strains are color-coded by location of collection according to the key. Labels “*i*” to “*ii*” denote examples of phylotype variation signatures referred to in the text. Label “*i*” shows a pattern of underrepresented *P1-3* among strains of multiple countries; “*ii*” shows a pattern of overrepresented *P5-6* in Gambian isolates; “*iii*” shows a pattern of underrepresented *P15* common to Forest-subtype isolates.

Although the relative proportions of VSG phylotypes appear to be a fixed feature of the *T. congolense* genome, they are not entirely invariant. When phylotype abundances are normalized by the cohort mean, subtle fluctuations in phylotype size are detected (Fig. 4). For example, there is a signature of underrepresented *P1-3* in samples from Kenya, Uganda, Tanzania, and Burkina Faso (IL3978 to IL3578, “i”), and the Gambian isolates show a combination of overrepresented *P5* and *P6* that is not observed elsewhere (“ii”). Also, the Forest-subtype isolates show a distinct underrepresentation of *P15* (“iii”). Furthermore, the degree of strain variation in phylotype abundance correlated with phylotype size itself, such that high-abundance phylotypes, i.e., with more genes (e.g., *P15*), are consistently more variable than low-abundance phylotypes (e.g., *P8* and *P9*; *R*^2^ = 0.74). We believe this variation reflects gene gain and loss within phylotypes on a population scale, which may have functional or clinical implications.

### The relationships between VSG repertoires are distinct from the population structure

To better understand what might explain the strain variation in the VAP, we examined the congruence of the VAP with population history. Whole-genome SNPs were called using GATK and analyzed using RAxML (Fig. 3A). Savannah and Forest subtypes are clearly separated (IL3900 and IL3926, “i”). Within the Savannah subtype, there is a geographical signature only toward the top of the phylogeny (IL274 to IL3304, “ii”). The remaining isolates do not recapitulate geography, particularly when looking at the short phylogenetic distance between IL3954 and IL1180 (“iii”) from Nigeria and Tanzania, respectively.

When comparing the VAPs of genetically close isolates (Fig. 3B), conserved patterns can be seen for some, but not all, groups. For instance, among seven Kenyan samples (IL274 to ILC55, “iv”), there is little variation in SNPs. However, ILC55 has a distinctive VAP; note the profusion of *P5-7* and the scarcity of *P1-3*. In the VAP dendrogram (Fig. 3C), ILC55 clusters with IL3932, IL588, IL2326, and IL2068, three of which are from a different population group and were isolated in different countries (i.e., Kenya, Uganda, and Tanzania). In another example, a Tanzanian strain, ILC22, is genetically close to a large clade of Kenyan strains (i.e., IL3349 to IL3775, “v”) but displays a genomic VSG repertoire like other Kenyan strains (i.e., IL274 to IL409, “iv”) due to lower numbers of *P7* and *P12*.

Although the possibility of labeling errors can never be ruled out, we are confident that the association between the VAP and population structure is genuinely weak. African trypanosome genomes include extended subtelomeric domains that contain large numbers of VSG and other multicopy gene families and are distinct from chromosomal “cores” containing conserved polycistrons of housekeeping genes (Horn and Barry 2005). These subtelomeres are held to be hemizygous (Callejas et al. 2006), and it may be that genetic variation segregating them is decoupled from diploid loci in chromosomal cores.

### VAP applied to metacyclic VSG expression identifies preferential expression of “rare” phylotypes

We extended antigen profiling to transcriptomic data to show how a combination of read mapping and structural motif searching can produce transcript abundance-weighted VAPs. We illustrate this by profiling the metacyclic-stage VSG repertoire (mVSG) of *T. congolense* extracted from experimentally infected tsetse mouthparts.

**Figure 5.**
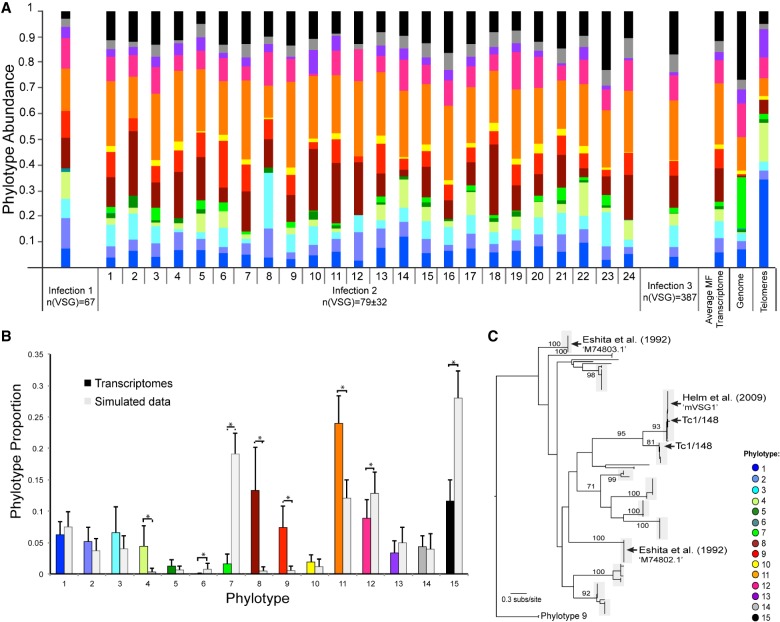
VAP applied to mVSG expression in experimentally infected tsetse mouthparts. (*A*) Transcriptomic VAPs of trypanosomes extracted from tsetse mouthparts. VAPs from the transcriptomes are remarkably similar yet significantly different from the genomic VSG repertoire (Poisson regression, *P* < 0.001) and the VSG found at telomeric expression sites. Infection 1 represents a sample of 40 pooled mouthparts; infection 2 represents 24 individual mouthparts; infection 3 represents a sample of 131 pooled mouthparts after metacyclic parasite enrichment by anion exchange chromatography. The genomic VAP represents the average profile of 24 sets of 79 VSGs randomly sampled from the genome of Tc1/148. Stacked columns are color-coded by phylotype according to the key. The number of VSG transcripts recovered in each sample infection is noted in the figure. (*B*) Comparison of average phylotype proportion (adjusted for transcript abundance) in transcriptomic samples presented in *A* and genomic profiles from a random selection of *VSG* of Tc1/148 (mean ± σ). Statistical analysis reveals that, in comparison to the genome, *P7*, *P12*, and *P15* are underrepresented in the transcriptomes (independent *t*-test, *P*-value <0.001), while *P4*, *P8*, *P9*, and *P11* are significantly overrepresented (independent *t*-test, *P*-value <0.001). (*C*) ML phylogeny of *P8* showing 12 distinct loci found across our *T. congolense* strain genomes (denoted by gray boxes), the position of Tc1/148 *P8* transcripts, and those from two previous studies (Eshita et al. [1992] [UniProt ID “M74803.1” and “M74802.1”] and Helm et al. [2009] [“mVSG1”]). Internal nodes are labeled with bootstrap values greater than 70.

We produced a transcriptome from 40 pooled mouthparts from tsetse infected with *T. congolense* strain 1/148 (Young and Godfrey 1983) to establish if sufficient RNA could be recovered to produce a reliable VAP. We recovered 67 VSG transcripts, relating to various phylotypes, although the single most abundant VSG transcript belonged to *P8* (Fig. 5A, infection 1). However, the information obtained from pooled data is limited because we cannot estimate the degree of variation between flies and thereby evaluate the reproducibility of the VAP. Hence, we produced transcriptomes from 24 individual tsetse flies infected with blood stabilates of the same *T. congolense* strain 1/148, recovered from infection 1 and passaged once through mouse. The transcriptomes contained 20.4–37.8 M reads per sample, of which 6%–47% mapped to the *T. congolense* 1/148 genome sequence. The mapped reads resulted into 6462–11,466 transcripts, of which 31–147 were VSGs (mean ± σ = 79 ± 31; FPKM = 103–634) (Supplemental Table S2). After profiling the VSG transcripts and weighting for transcript abundance, remarkably low variation is seen among flies, but the VAP itself is quite distinct from the genomic profile (Fig. 5A, infection 2).

To confirm the significance of the difference between transcriptomic and genomic VAPs, we created simulated VAPs to investigate whether any specific phylotypes were under- or overrepresented in the transcriptomes given their frequencies in the genome. As shown in Figure 5B, the transcriptomic VAPs are consistently distinct from the genomic repertoire (Poisson regression, *P* < 0.001). The proportions of seven phylotypes were significantly different among mVSGs relative to the genome (Fig. 5B): *P7*, *P12*, and *P15* are underrepresented in the transcriptomes (independent *t*-test, *P*-value <0.001), while *P4*, *P8*, *P9*, and *P11* are significantly overrepresented (independent *t*-test, *P*-value <0.001) (Fig. 5B). This indicates that those overrepresented phylotypes may be preferentially expressed in the metacyclic stage.

A closer analysis of *P11* and *P8*, the most abundant and the most overrepresented in the metacyclic transcriptomes, respectively, reveals major differences in composition. *P11* includes 146 genes, of which 74 (50%) are expressed across infections; these display variable (but generally low) transcript abundances (FPKM = 8 × 10^−5^ to 70). Only one transcript (78% identical to TcIL3000_0_57360) is found in all infections, while 29 are infection specific. This suggests the abundance of *P11* relates to the phylotype generally and not to any specific gene. Conversely, the relative abundance of *P8* derives from two transcripts common to all infections (98% and 99% identical to TcIL3000_0_09520, respectively) and a third transcript common to 23/24 samples (99% identical to TcIL3000_5_650). These three transcripts have consistently high expression values (sum FPKM per infection: 82.68 to 639.16). In fact, in 21/24 individual fly transcriptomes, they are among the six most abundant VSG transcripts (Supplemental Fig. S3). Thus, in contrast to *P11*, the abundance of *P8* seems due to reproducible expression of specific genes; the position of these within *P8* is shown in Figure 5C.

Finally, as tsetse mouthparts contain parasites in multiple developmental stages, each potentially expressing VSG at low levels that could affect the profile, we estimated VAPs from metacyclic-enriched populations obtained from a third fly infection. The nonenriched mouthpart parasite population was predominantly composed of epimastigotes and other nonmetacyclic intermediate forms (up to 82%). Metacyclic parasites were selected by anion exchange chromatography using a DE52 cellulose column, which resulted in a parasite population composed of up to 76% metacyclic forms. The VAP of the enriched population was not significantly different to those of the nonenriched (*R*^2^ = 0.83, *P* < 0.001), showing that the VAP faithfully reflects metacyclic-form gene expression even when those are a small proportion of the total cells in the fly mouthparts. Using anion exchange chromatography to select metacyclics could theoretically result in VSG selection by charge. However, we see no significant differences in VSG expression between this transcriptome and those from infections 1 and 2, where no selection or enrichment was done, so we are confident that we have introduced no artifact.

The VAPPER processes raw genomic or transcriptomic sequencing reads and produces antigen profiles expressed in multiple formats (i.e., table of frequencies, heatmaps, and PCA plots), placing the profile in the context of *T. congolense* genomic isolates included here and previously published (Tihon et al. 2017).

## Discussion

We have described the VAP, a bioinformatic approach to describing the complete VSG repertoire of any *T. congolense* strain from genomic or transcriptomic data. We show how the VAP can be applied to the dynamics of VSG diversity among clinical isolates and in functional experiments to answer fundamental questions in parasite biology, i.e., the preferential expression of specific VSG in *T. congolense* metacyclic forms.

VAP will become most powerful when, and if, phylotypes become associated with distinct functions or phenotypes. We think this is plausible because our previous analysis of VSG phylogeny suggested that *T. congolense* IL3000 phylotypes are ancestral features, which do not recombine with each other and which, in some cases, predate the origin of *T. congolense* itself (Jackson et al. 2012). Our data corroborate this view by showing that the 15 phylotypes are universal among strains; the fact that *T. congolense* VSG segregate into 15 conserved clades is consistent with functional differentiation within the repertoire. If VSG phylotypes are functionally distinct, we would expect these differences to be preserved by purifying selection. We examined this, and individual VSGs appear to be under purifying selection comparable to the genomic background (ω (*d*_N_/*d*_S_) < 1). We calculated ω (*d*_N_/*d*_S_) for orthologous VSG in different strains and found an average of 0.27 (*N* = 1034), apart from *P8-10*, which is not significantly different from the average ω for single-copy orthologs across the genome (0.19; *N* = 694; *P* > 0.05). Only *P8-10* showed any deviation toward a more neutral substitution rate (0.73; *N* = 123).

In addition to conservation of the structural distinctions between phylotype sequences, we also observe that the relative proportions of each phylotype remain consistent across the population. In fact, the cladistic composition of the genomic repertoire is essentially a fixed feature of *T. congolense* Savannah (and is not substantially altered in the Forest subtype either). This might be surprising given the obvious pressures to diversify VSG repertoires in the population. If the different phylotypes were functionally redundant and existed simply to increase VSG structural diversity, we might expect individual phylotypes to fluctuate in size according to a random gene birth-and-death process. Instead, our results suggest persistent negative selection on gene gain and loss. Neutral evolution of VSG copy number would also result in greater variation among low-abundance phylotypes. If there were random fluctuations in VSG complement, we would expect phylotypes with a few genes, e.g., *P8* (*N* = 12), to be entirely absent in some strains. Yet, we observe the opposite statistical effect; low-abundance phylotypes are the least variable among strains when abundance is corrected for size. This suggests that, while fluctuation in high-abundance phylotype copy number is tolerable, low-abundance phylotypes are essential over evolutionary timescales.

Thus, we consider the discrete VSG phylotypes, the negative selection on their sequences, their stable proportions in the genome, and the persistence of rare forms to be features consistent with functional differentiation among *T. congolense* VSGs. This idea is supported by the several *T. brucei* VSGs that have acquired new functions. The transferrin receptor gene family, required for parasite uptake of host transferrin, is derived from a-type VSGs in both *T. brucei* and *T. congolense* (Salmon et al. 1997). *ESAG2* derives from b-type VSGs (Jackson et al. 2012) but is now antigenically invariant and localized in the flagellar pocket of bloodstream forms (Gadelha et al. 2015). The *SRA* and *tgsGP* genes, required for human infectivity, are derived from a-type and b-type VSGs, respectively (De Greef and Hamers 1994; Van Xong et al. 1998; Berberof et al. 2001; Capewell et al. 2013; Uzureau et al. 2013). Finally, a recent example suggests that suramin resistance in *T. brucei* is associated with neofunctionalization of a specific *VSG* gene (Wiedemar et al. 2018).

Functional differentiation is also seen in *P. falciparum var* genes (Gardner et al. 2002; Kraemer and Smith 2003; Lavstsen et al. 2003). Our profiling approach is similar to how *var* gene antigenic diversity is measured using a population genomic framework and the cumulative diversity of the conserved Duffy binding like alpha (DBLα) domain (Barry et al. 2007). Specific group A *var* genes have been reproducibly linked to disease severity (Kirchgatter and Del Portillo 2002; Bull et al. 2005; Kyriacou et al. 2006; Wang et al. 2012). Moreover, the atypical *var2csa* gene, unique for retaining orthology across *P. falciparum* strains, may play a regulatory role in the expression of other family members (Ukaegbu et al. 2015; Bryant et al. 2017).

Hence, we have circumstantial evidence of functional differences among *T. congolense* VSG, made plausible by differentiation among comparable variant gene families. VSG phylotypes might be expressed in specific developmental stages, tissues, hosts, or syndromes, and we believe that VAP will be instrumental in exposing such phenotypic differences in transcriptomic data from natural and experimental infections. In *T. brucei* bloodstream-form parasites, such experiments have revealed a surprising level of VSG transcript diversity during infections (Hall et al. 2013; Mugnier et al. 2015), challenging the dogma that each growth peak is essentially associated with a single VSG. This may be true for *T. congolense* also, and this study provides a rational approach to VSG expression dynamics in future experiments.

This study has begun to explore functional differentiation by profiling VSG expression in the metacyclic stage, a developmentally distinct stage to bloodstream forms. Among metacyclic forms in the same fly, multiple VSGs are expressed in comparable abundance; with observed repertoires of 15 (or less) and 27 antigen types in *T. congolense* and *T. brucei*, respectively (Esser et al. 1982; Crowe et al. 1983; Lenardo et al. 1986; Turner et al. 1988). This is quite different from the situation in bloodstream forms where one or two superabundant VSG isoforms are expressed at any given time (Helm et al. 2009). As in previous studies, we asked whether mVSGs are a random selection of available variant antigens or are a particular set of VSGs. We find that metacyclic VSG transcription in strain 1/148 is nonrandom and reproducible over time, having survived a full transmission cycle. *P8* is consistently overrepresented in metacyclic transcriptomes, and *P8* members are always among the most abundant VSG transcripts (Supplemental Fig. S3). Preferential expression of *P8* is corroborated by an earlier study of mVSG protein expression in *T. congolense* IL3000/ILNaR2 (Eshita et al. 1992) and sequence data from an EST library of *T. congolense* IL3000 in vitro metacyclics (Helm et al. 2009). Therefore, our evidence points to *P8* being preferentially expressed in the metacyclic stage and, so, possibly developmentally regulated.

It remains to be shown that *P8* is restricted to metacyclics or enriched in natural fly infections. However, the evidence thus far highlights a difference between mVSG expression between species. In *T. brucei*, mVSGs are randomly selected from the genomic repertoire and change over time in both natural infections and sequential laboratory tsetse transmissions of the same parasite clone (Barry et al. 1983). In *P. falciparum*, the *var* gene expression radically changes following a single mosquito passage (Bachmann et al. 2016). In *Plasmodium chabaudi*, vector passaging not only alters *cir* (chabaudi interspersed repeats) expression in the erythrocytic cycle but also leads to virulence attenuation, related to the broad activation of most subtelomeric variants (Spence et al. 2013). If the pattern of *T. congolense* mVSG expression is reproducible in nature, then the preferential expression of *P8* indicates a form of developmental regulation that may be exploitable in vaccine design.

Further research will also be needed to understand population variation in the VAP. We had expected to see a strong geographical signature in the VSG repertoire, based on other organisms (e.g., *var* diversity in natural *P. falciparum* populations) (Chen et al. 2011). However, the VAP overlaps in strains across Africa and is not strongly geographically defined in our sample at least. This suggests that variation in VSG repertoire is decoupled from global population history as inferred from genome SNPs. This may simply reflect our nonsystematic strain sample or, indeed, errors in sample labeling. To assess this, we profiled 52 additional *T. congolense* strains from a recent study by Tihon et al. (2017), but relationships among these VAPs continue to conflict with population history and remain only partially explained by geography (Supplemental Fig. S4). The lack of concordance between SNPs and VAPs could result from asymmetric sorting of VSG during meiosis, i.e., if the hemizygous subtelomeres and mini-chromosomes upon which VSG loci are found are inherited in a non-Mendelian fashion. There is evidence that *T. congolense* is sexual and undergoes meiosis (Morrison et al. 2009; Tihon et al. 2017), as well as evidence of gene flow between *T. congolense* populations in the form of putative hybrid West African parasites that were found to circulate in Zambian populations (Tihon et al. 2017). Ultimately, the plausibility of sexual assortment of VSG repertoires independent of other markers will need to be tested in experimental crosses, but it remains possible that the VAP may be a useful epidemiological marker with unique characteristics.

VAP could be applied to other African trypanosome species, but each species would require a bespoke approach to the peculiarities of its antigenic repertoires. A motif-based approach for Trypanosoma *vivax*, which has many more phylotypes in lower copy number (Jackson et al. 2012), will be described in a forthcoming publication (S Silva Pereira and A Jackson, unpubl.). A *T. brucei* VAP must contend with pervasive sequence mosaicism due to recombination and an absence of stable phylotypes that could be discriminative (Marcello and Barry 2007; Hall et al. 2013); thus, it is likely that motif combinations will be more informative than simple frequencies in this case.

In conclusion, we can accurately profile VSG repertoires from *T. congolense* genomes and transcriptomes. We anticipate that individual VSG phylotypes are functionally differentiated and that VAP will help in revealing these differences. Ultimately, by associating individual phylotypes with distinct functions, such as developmental stages, pathology, or host use, we can reveal the relationship between disease and VSG variation, and the VAP could become an important diagnostic and epidemiological marker. Variant antigens have long been described as intricately involved in virulence and pathology but highly dynamic and refractory to analysis en masse. This study has revealed the scale of global antigenic diversity in *T. congolense* and provided the first approach to its high-throughput analysis in population and experimental settings.

## Methods

### Sample preparation and genome sequencing

#### Field isolates

A panel of 41 *T. congolense*-infected blood stabilates (150 µL), representing isolates from Burkina Faso (*N* = 5), Kenya (*N* = 23), Nigeria (*N* = 3), Tanzania (*N* = 5), The Gambia (*N* = 3), and Uganda (*N* = 2), were selected from the Azizi Biorepository (http://azizi.ilri.org/repository/) at the International Livestock Research Institute (Supplemental Table S1).

Parasite DNA was enriched by depleting host leukocytes in the whole-blood stabilates using anti-CD15 (no. 130-094-530, Miltenyi Biotec) and anti-CD45 antibodies (no. 130-052-301, Miltenyi Biotec), as most leukocytes have one or both antigens. DNA samples were sequenced on the Illumina MiSeq platform as 150- or 250-bp paired ends. A detailed protocol for the enrichment and further details on genome sequencing and assembly are provided in Supplemental Methods.

### VSG-like sequence recovery, alignment, and analysis

VSG-like nucleotide sequences were manually retrieved from the assembled contigs files. To recover all VSG-like sequences in the genomes, a sequence similarity search was performed with tBLASTx using a database of *T. congolense* IL3000 VSG as query and a significance threshold of *P*-value >0.001, contig length greater than 150 amino acids, and percentage identity of 75 or more. Sequences with 40%–75% similarity to the reference were manually inspected and its inclusion in the analysis empirically decided. Recovered sequences were assigned one of 15 VSG phylotypes based on their best match in the IL3000 reference sequence. Phylotype relative frequencies were used to manually estimate VAPs, which were subsequently compared with motif-based profiles.

VSG-like sequences were translated with BioEdit 7.2.5 (Hall 1999) and aligned with ClustalW (Larkin et al. 2007). For each strain, a VSG phylogeny was estimated from a protein sequence alignment of recovered VSG-like sequences and IL3000 VSG sequences with the neighbor-joining (NJ) method and the WAG+Γ substitution model (Whelan and Goldman 2001) using MEGA7 (Kumar et al. 2016). All full-length VSG sequences from IL3000, IL3674 (The Gambia), and IL3900 (Burkina Faso, Forest subtype) were aligned with ClustalW (Larkin et al. 2007) to produce a VSG phylogeny representative of the *T. congolense* species. Further details are described in Supplemental Methods.

### VSG phylotyping

Taking sequence alignments for each phylotype, we used a heuristic process to identify strings of nine to 59 amino acids that were uniquely diagnostic of each phylotype. Twenty-eight motifs were identified that, collectively, reproduced the known phylotype frequencies of the full-length, reference VSG repertoire (*N* = 593) (Supplemental Fig. S1). Further information on phylotype motif development and validation is provided in the Supplemental Methods and Supplemental Figures S5 and S6. Motif screening of assembled contigs was performed with HMMER3 under default parameters (Eddy 2009), and the relative frequencies of each phylotypes were used to create the automated VAP. To compare the stability of the composition of the VAPs to the background random variation, the total pool of VSGs recovered in the study was used to create 41 randomized, simulated VAPs containing 250 VSGs each.

### Tsetse fly infection and rearing

For *T. congolense* Savannah 1/148 (MBOI/NG/60/1-148) (Young and Godfrey 1983), Tc1/148 mouse blood stabilates were obtained from the Department of Parasitology of the Liverpool School of Tropical Medicine, UK, and cultured on modified Eagle's medium (MEM)–based modified differentiating trypanosome medium (DTM) (10% fetal bovine serum, 2 mM L-glutamine, 10 mM L-proline and no glucose) and 0.5 mg/mL penicillin/streptomycin at 27°C, 5% CO_2_. Experimental teneral (12–48 h post-eclosion) male tsetse flies (*Glossina morsitans morsitans*) were infected at the first blood meal Tc1/148 procyclic or bloodstream forms in sterile defibrinated horse blood supplemented with 10 mM glutathione via a silicone membrane as previously described (Moloo 1971). Flies were killed by decapitation and dissected at day 28 post-infection (p.i.) according to the description of Peel (1962). Further details on the methods used are described in the Supplemental Methods.

### RNA extraction and sequencing

#### Infections 1 and 2

Total RNA from hypopharynx dissections were extracted with the AllPrep RNA/protein kit (Qiagen) according to the manufacturer's protocol, yielding RNA outputs of 48–213 ng per sample.

#### Infection 3

RNA was extracted using the RNeasy kit (Qiagen), yielding a total RNA output of between 48 and 246 ng. In both cases, RNA-seq libraries were prepared at Centre of Genomic Research (Liverpool, UK) using the NEBNext Ultra II Directional RNA library prep kit with poly(A) selection (Poly(A) mRNA magnetic isolation module) (New England Biolabs) as per the standard procedure. RNA-seq libraries were sequenced on the HiSeq 2500 platform (Illumina) as 150 paired ends, producing 280 million mappable reads.

### Transcriptome profiling

RNA-seq reads were mapped to the tsetse fly genome (International *Glossina* Genome Initiative 2014) to deplete host reads using Bowtie 2 (Langmead and Salzberg 2012), and the unmapped data were mapped to the *T. congolense* IL3000 genome. Transcript abundance values were estimated from the BAM file using Cufflinks (Trapnell et al. 2012). VSG transcripts and abundance values (FPKM) were extracted from the Cufflinks output, screened for the phylotype motifs described previously. Transcriptomic VAPs were estimated by adjusting the phylotype frequency for the relative combined abundance of all transcripts in a given phylotype. To test if the observed VSG transcriptome was a random sample of the genomic repertoire, 24 randomized VAPs (i.e., one for each transcriptome in infection 2) were simulated by sampling 79 sequences (i.e., mean number of VSG in observed transcriptomes) from a VSG pool derived from all strain genome sequences.

### Statistical analysis

The statistical comparisons between the BLAST and the VAP performances in recovering VSGs were done using the Pearson's correlation test. Outliers were identified using a threshold of 2xσ with the function “removeOutlier” in R (R Core Team 2017). Outliers were manually inspected before removal. The statistical analyses of differential profile expression used the Poisson regression model; *F*-tests were performed to analyze variance between observed and simulated data both from transcriptomic and genomic data; and independent Student's *t*-tests were performed to detect statistical significances in phylotype relative abundances. All tests were performed in R (R Core Team 2017).

## Data access

The data generated as part of this study have been submitted to the NCBI BioProject database (https://www.ncbi.nlm.nih.gov/bioproject) under accession numbers PRJNA387239 and PRJNA399822 and to the European Nucleotide Archive (ENA; https://www.ebi.ac.uk/ena) under accession number ERP023223. The VAPPER pipeline has been compiled in a Python script that is available from GitHub (https://github.com/johnheap/Trypanosoma-VAP) and in Supplemental File S1.

## Supplementary Material

Supplemental Material
