# Supplementary material for: Variant antigen repertoires in Trypanosoma congolense populations and experimental infections can be profiled from deep sequence data using universal protein motifs
Source: Genome Res. 2018 Sep;28(9):1383–94. doi: 10.1101/gr.234146.118 (PMC6120623; doi:10.1101/gr.234146.118)
Supplement: Supplemental Material [file supp_28_9_1383__index.html]

Variant antigen repertoires in Trypanosoma congolense populations and experimental infections can be profiled from deep sequence data using universal protein motifs — Supplemental Material 

# Variant antigen repertoires in *Trypanosoma congolense* populations and experimental infections can be profiled from deep sequence data using universal protein motifs

## Supplemental Material

- Supplemental\_Material.zip
