# Supplementary material for: Variant antigen repertoires in Trypanosoma congolense populations and experimental infections can be profiled from deep sequence data using universal protein motifs
Source: Genome Res. 2018 Sep;28(9):1383–94. doi: 10.1101/gr.234146.118 (PMC6120623; doi:10.1101/gr.234146.118)
Supplement: Supplemental Material [file supp_gr.234146.118_Supplemental_Material.zip › Supplemental_Material/Supplemental_Methods.pdf]

Host cell depletion and DNA extraction

Parasite DNA was enriched by depleting host leukocytes in the whole blood stabilates using anti-CD15 (#130-094-530, Miltenyi Biotec, UK) and anti-CD45 antibodies (#130-052-301, Miltenyi Biotec, UK), as most leukocytes have one or both antigens. Red blood cells were lysed with ACK lysing buffer (0.15M NH_4Cl, 10mM $KHCO_3$, 0.1mM EDTA) and discarded by centrifugation. The remaining cell pellet was washed with 500µl MACS buffer (2mM EDTA, 5xBSA in PBS pH7.2) incubated with 10µl of mouse anti-$CD15^+$ (#130-094-530, Miltenyi Biotec, UK) and 10µl of anti-$CD45^+$ (#130-052-301, Miltenyi Biotec, UK) for 15 minutes at 4˚C. Cells were washed in 1ml MACS buffer by centrifugation (10 minutes, 2500rpm) and the pellet resuspended in 500µl MACs buffer. The cell suspension was passed through a previously washed magnetic MACS column. The first eluate was collected, centrifuged for 10 minutes at 2500rpm and the pellet resuspended in 100µl lysis buffer (aqueous solution of 1M Tris-HCl pH8.0, 0.1mM NaCl, 10µM EDTA, 5% SDS, 0.14µM Proteinase K). Samples were incubated at room temperature for 1 hour and DNA was extracted with magnetic Sera-Mag Speedbeads (GE Healthcare Life Sciences, UK) according to the manufacturer's protocol. Eleven samples were subject to genomic amplification to increase the DNA output using the illustra GenomiPhi V2 DNA Amplification Kit (GE Healthcare Life Sciences, UK) according to the manufacturer's protocol (samples IL274, IL374, IL396, IL409, IL439, IL3022, IL3296, IL3775, IL3900, IL3954 and ILC22). DNA outputs used for sequencing ranged from 4ng to 123 ng (unamplified samples) and 14ng to 1.1µg (amplified samples).

Genome sequencing and assembly

Illumina paired-end sequencing libraries were prepared from genomic DNA using the Accel-NGS 2S DNA Library Kit (Swift Biosciences, Inc., USA) and sequenced by standard procedures on the Illumina MiSeq platform, as 150 or 250bp paired ends (SRA accession no. ERP023223). For each sample, the data yield from sequencing after quality filtering was between 6.19×10^5 and 1.33×10^7 read pairs. These were assembled *de-novo* using Velvet (Zerbino & Birney 2008) with the following parameters: kmer of 65, insert length adjusted for 400 base pairs with standard deviation of 50, and minimum pair count of 20. These produced assemblies with n50 between 161 and 3257 base pairs (median = 700). Allele frequencies were inspected to ensure samples were from single infections only.

VSG-like sequence alignment and phylogenetic estimation

All full-length VSG sequences from IL3000, IL3675 (The Gambia), and IL3900 (Burkina Faso, Forest sub-type) were aligned with ClustalW (EBI, UK) (Larkin et al. 2007) to produce a VSG phylogeny representative of the *T. congolense* species. The alignment contained

1037 sequences in total, 778 from IL3000, 214 from IL3675, 31 from IL3900, 12 *T. brucei* ESAG2 and 2 *T. vivax* b-type VSG as the outgroup. The representative VSG phylogeny was estimated from protein sequence alignments with the maximum likelihood (ML) method and the WAG+Γ substitution model (Whelan and Goldman 2001) using RAxML v.2 (Stamatakis 2014) and PhyML (Lefort et al. 2017) following amino acid model selection in PhyML (Lefort et al. 2017). Robustness was assessed with 100 bootstrap replicates. Bayesian inference (BI) trees were estimated with gamma rates function in MrBayes v3.1.2. (Huelsenbeck and Ronquist 2001; Ronquist and Huelsenbeck 2003) and two Markov chain Monte Carlo chains run in parallel over 5,000,000 generations, with a burnin of 2500 and a fixed WAG+Γ model to ensure the program would reach stationary convergence. Posterior probabilities of each node were used to assess accuracy of BI trees. Neighbor-Joining (NJ) trees were estimated in PHYLIP v3.2 (Felsenstein 1989) using the executables protdist and neighbor for a multiple dataset of 100 bootstrap replicates and a random seed of 99. Maximum likelihood rations of each clade were calculated and compared with RAxML (Stamatakis 2014) in triplicate. For each strain and clade, a VSG was randomly chosen and forced to cluster in the adjacent clade and negative log-likelihood of unconstrained trees was compared to that of constrained trees.

Phylotype motif development and validation

To identify protein motifs diagnostic of each phylotype, we have used a protein sequence alignment containing all VSG sequences recovered from all strains. As we did not know whether the IL3000 VSG repertoire would be a good predictor of other *T. congolense* strains, we used a low threshold for sequence similarity (40%), for recovering VSG sequences by sequence similarity search (tBLASTx). This is the similarity seen when we compare a *T. congolense* VSG with a *T. brucei* or a *T. vivax* b-type VSG. Therefore, this threshold would theoretically capture any previously unseen VSG-like sequence. The length threshold applied (i.e. 150 amino acids) was chosen to avoid spurious matches and very short contigs during the initial phylogenetic analysis. This length represents approximately half of the *T. congolense* VSG sequence, which would allow a reliable multiple sequence alignment and placement in the phylogeny. Both these thresholds were used for the VSG alignments used for motif design only and they do not affect subsequent profiling (which uses all fragments larger than the motifs themselves).

We have identified the motifs by looking at the alignment and spotting strings of sequences that looked specific for that particular phylotype. These tentative motifs were tested on a set

of full-length VSGs from the IL3000 genome sequence. This set of sequences worked as a positive control for motif validation because the frequencies of each phylotype were previously known by manual calculation. Motifs were trimmed, extended, or combined with other protein signatures until they could accurately identify all sequences from their particular phylotype in the positive control. A final set of 28 motifs that provided an accurate VAP for the IL3000 full-length reference VSGs was chosen for the VAPPER (Supplemental Figure S1).

To evaluate the robustness of the VAPPER for profiling poorly assembled (i.e. highly fragmented) and incomplete genomes, we performed a series of simulations. To assess the effect of fragmentation in profiling, we split each VSG into subsequences of increasingly smaller sizes. We started at 90% (i.e. 999 nucleotides) of the average length of the VSG sequences present in the IL3000 positive control (i.e. 1163 nucleotides) and decreased the lengths to 20% (i.e. 44 nucleotides) in 10% intervals. These new sequence files were used to calculate VAPs and the correlations between the relative phylotype frequencies of the full and the fragmented sequences were calculated (Supplemental Fig. 5). To assess the effect of missing data, we profiled randomly selected, increasingly smaller percentages of the IL3000 positive control and the correlations between the relative phylotype frequencies of the full and the incomplete repertoires were calculated (Supplemental Fig. 6).

Strain variation

To estimate strain relationships based on the whole genome, MiSeq reads were retrieved and mapped against the *T. congolense* IL3000 genome using BWA-MEM (Li 2013), converted to BAM format, sorted and indexed with SAMtools (Li et al. 2009). Sorted BAM files were cleaned, duplicates marked and indexed with Picard (http://broadinstitute.github.io/picard/), and SNPs were called and filtered with Genome Analysis Toolkit suite according to the best practice protocol for multi-sample variant calling (Van der Auwera et al. 2013). The multi-sample vcf file obtained from GATK was converted to fasta format using VCFtools v0.1.14 (Danecek et al. 2011) and a maximum likelihood phylogeny was estimated with RAxML, using the JTT+Γ model of nucleotide substitution, following nucleotide model selection on MEGA7 (Kumar et al. 2016). The SNP analysis of the combined data including the samples published by Tihon et al. (2017) presented in Supplemental Fig. S4 was performed under the same conditions.

Tsetse fly infection and rearing

Infection 1: Experimental teneral (12-48h post-eclosion) male tsetse flies (*Glossina morsitans morsitans*) were infected at the first blood meal with 5x10^5 per ml^{-1} of Tc1/148

procyclic forms in sterile defibrinated horse blood supplemented with 10mM glutathione via a silicone membrane as previously described (Moloo 1971). One day after infection, unfed flies were sorted and removed. Remaining flies were maintained at 26˚C (+/-1˚C) and 65-75% relative humidity and fed every 2-3 days with normal sterile defibrinated horse blood. Flies were killed by decapitation at day 28 post-infection (p.i.) and mouthparts dissected according to the description of Peel (1962). When metacyclic parasites were visible, the hypopharynx and the parasite suspension that was released during dissection were collected and frozen in liquid nitrogen.

Infection 2: A frozen stabilate of 1ml of Tc1/148 infected mouse blood (20% glycerol and parasitaemia of 10^3 parasites/ml) was thawed and mixed with defibrinated horse blood at a 1:10. The sample was used to infect 100 teneral male tsetse flies (*G. m. morsitans*) as described in the previous section. Flies were killed and dissected at day 28 post-infection (p.i.) as described above.

Infection 3: Experimental teneral male tsetse flies (*G. m. morsitans*) were infected 12-48h post-eclosion with $5x10^5$ per ml^{-1} Tc1/148 procyclic forms in sterile defibrinated horse blood supplemented with 10mM glutathione, and reared according to the procedure described in the previous section. Flies were killed by decapitation at 29 days p.i. into a drop of glucose separation buffer (44mM NaCl, 57mM Na_2HPO_4, 3mM KH_2PO_4, 55mM glucose, pH 8.0 at 20ºC) and visualized under the light microscope (10X magnification). Dissections were performed according to the description of Peel (1962). When metacyclic parasites were visible by microscopy, the hypopharynx was broken down to release the parasites and the parasite suspension collected by aspiration and kept on ice for metacyclic parasite separation by anion exchange chromatography using DE52 cellulose, as previously described (Lanham and Godfrey 1970). To prepare the columns, 200μl DE52-cellulose was added to a Poly-Prep Chromatography Column (Bio Rad, UK) and allowed to settle. Columns were equilibrated with 400μl glucose separation buffer (44mM NaCl, 57mM Na_2HPO_4, 3mM KH_2PO_4, 55mM glucose, pH 8.0 at 20ºC) by gravity to remove chloroform. 50ul of trypanosome mixture was added to the column and eluate recovered on ice. Column was washed with 5 volumes of glucose separation buffer and eluate recovered on ice. Eluate was checked by microscopy and metacyclic:epimastigotes ratio calculated by hemocytometry and DAPI staining of a subset of purified parasites. Parasites were snap frozen on liquid nitrogen and kept at -80ºC until RNA and protein extractions.

Methods References:

Danecek P, Auton A, Abecasis G, Albers CA, Banks E, DePristo MA, Handsaker RE, Lunter G, Marth GT, Sherry ST, et al. 2011. The variant call format and VCFtools. *Bioinformatics* **27**: 2156–2158.

Felsenstein J. 1989. PHYLIP - Phylogeny inference package - v3.2. *Cladistics* 164–166.

Huelsenbeck JP, Ronquist F. 2001. MRBAYES: Bayesian inference of phylogeny. *Bioinformatics* **17**: 754–755.

Kumar S, Stecher G, Tamura K. 2016. MEGA7: Molecular Evolutionary Genetics Analysis version 7.0 for bigger datasets. *Mol Biol Evol* **33**(7): 1870-1874

Lanham SM, Godfrey DG. 1970. Isolation of salivarian trypanosomes from man and other mammals using DEAE-cellulose. *Exp Parasitol* **28**: 521–534.

Larkin MA, Blackshields G, Brown NP, Chenna R, Mcgettigan PA, McWilliam H, Valentin F, Wallace IM, Wilm A, Lopez R, et al. 2007. Clustal W and Clustal X version 2.0. *Bioinformatics* **23**: 2947–2948.

Lefort V, Longueville J-E, Gascuel O. 2017. SMS: Smart Model Selection in PhyML. *Mol Biol Evol* 4–6.

Li H. 2013. Aligning sequence reads, clone sequences and assembly contigs with BWA-MEM. *arXiv Prepr arXiv* **0**: 3.

Li H, Handsaker B, Wysoker A, Fennell T, Ruan J, Homer N, Marth G, Abecasis G, Durbin R. 2009. The Sequence Alignment/Map format and SAMtools. *Bioinformatics* **25**: 2078–2079.

Moloo SK. 1971. An artificial feeding technique for *Glossina*. *Parasitology* **63**: 507–512.

Peel E. 1962. Identification of metacyclic trypanosomes in the hypopharynx of tstse flies, infected in nature or in the laboratory. *Trans R Soc Trop Med Hyg* **56**: 339–341.

Ronquist F, Huelsenbeck JP. 2003. MrBayes 3: Bayesian phylogenetic inference under mixed models. *Bioinformatics* **19**: 1572–1574.

Stamatakis A. 2014. RAxML version 8: A tool for phylogenetic analysis and post-analysis of large phylogenies. *Bioinformatics* **30**: 1312–1313.

Tihon E, Imamura H, Dujardin J-C, Van Den Abbeele J, Van den Broeck F. 2017. Discovery and genomic analyses of hybridization between divergent lineages of *Trypanosoma congolense*, causative agent of Animal African Trypanosomiasis. *Mol Ecol*. **26**: 6524–6538.

Van der Auwera GA, Carneiro MO, Hartl C, Poplin R, del Angel G, Levy-Moonshine A, Jordan T, Shakir K, Roazen D, Thibault J, et al. 2013. From fastQ data to high-confidence variant calls: The genome analysis toolkit best practices pipeline. *Curr Protoc Bioinforma*.
