# Supplementary material for: Variant antigen repertoires in Trypanosoma congolense populations and experimental infections can be profiled from deep sequence data using universal protein motifs
Source: Genome Res. 2018 Sep;28(9):1383–94. doi: 10.1101/gr.234146.118 (PMC6120623; doi:10.1101/gr.234146.118)
Supplement: Supplemental Material [file supp_gr.234146.118_Supplemental_Material.zip › Supplemental_Material/Supplemental_Table_S1.pdf]

Supplemental Table S1 Description of samples used in the study, their sequencing and assembly statistics.

Sample ID	Year	Host	Passage Species	Country	Location	Number of reads	n50
**IL1180**	1961	Lion	Mouse	Tanzania	Serengeti	1.16E+07	207
**IL2068**	1971	unknown	Mouse	Tanzania	Serengeti	6.80E+05	1977
**IL2281**	1979	Cattle	Mice	Nigeria	Zaria	1.33E+07	1075
**IL2326**	1962	Cattle	Mice	Uganda	Uganda	8.18E+05	1599
**IL274**	1976	Dog	Mice	Kenya	Kabete	2.19E+06	611
**IL2992**	1966	Cattle	Mice	Kenya	Transmara	2.14E+06	1114
**IL2995**	1983	Cattle	Rat	Burkina Faso	Bobo Upper Delta	2.91E+06	223
**IL3019**	1966	Cattle	Mice	Kenya	Transmara	2.22E+06	1072
**IL3021**	1966	Cattle	Mice	Kenya	Transmara	1.87E+06	1089
**IL3022**	1966	Cattle	Mice	Kenya	Transmara	3.06E+06	633
**IL3035**	1985	Cattle	Mice	Kenya	Muhaka	2.26E+06	609
**IL311**	1979	Cattle	Rat	The Gambia	unknown	8.33E+05	3257
**IL3180**	1966	Cattle	Mice	Kenya	Transmara	1.57E+06	1550
**IL3296**	1972	Cattle	Mice	Tanzania	Robanda	1.68E+06	889
**IL3304**	1967	Cattle	Mice	Nigeria	Zaria	8.11E+05	397
**IL3349**	1966	Cattle	Mice	Kenya	Transmara	8.37E+05	993
**IL3578**	1983	Cattle	Rat	Burkina Faso	Bobo Upper Delta	6.83E+05	161
**IL3674**	1979	Cattle	Rat	The Gambia	unknown	3.37E+06	1113
**IL3675**	1979	Cattle	Rat	The Gambia	unknown	3.24E+06	1615
**IL3686**	1982	Lion	Mice	Kenya	Kenya	2.24E+06	190
**IL3688**	1982	Lion	Mice	Kenya	Kenya	2.86E+06	912
**IL374**	1976	Dog	Mice	Kenya	Kabete	1.63E+06	1217
**IL3775**	1966	Cattle	Rat	Kenya	Transmara	2.95E+06	1133
**IL3779**	1991	Tsetse fly	Mice	Kenya	Nguruman	6.19E+05	324
**IL3897**	1982	Cattle	Mice	Burkina Faso	Bobo Upper Delta	4.08E+06	528
**IL3900**	1980	Dog	Mice	Burkina Faso	Bobo Upper Delta	6.09E+06	314
**IL3926**	1980	Dog	Mice	Burkina Faso	Bobo Upper Delta	2.34E+06	235
**IL3932**	1992	Horse	Goat	Kenya	Delmonte	2.22E+06	930
**IL3949**	1972	Cattle	Cattle	Kenya	Taita	2.03E+06	665
**IL3954**	1967	Cattle	Mice	Nigeria	Zaria	4.90E+06	271
**IL396**	1976	Dog	Mice	Kenya	Nairobi	4.05E+06	736
**IL3978**	1992	Cattle	Mice	unknown	unknown	5.53E+06	511
**IL399**	1976	Dog	Mice	Kenya	Kabete	2.97E+06	741
**IL409**	1976	Dog	Mice	Kenya	Kabete	4.75E+06	361
**IL410**	1976	Dog	Mice	Kenya	Kabete	2.18E+06	955
**IL438**	1976	Dog	Mice	Kenya	Kabete	9.49E+05	418
**IL439**	1976	Dog	Mice	Kenya	Kabete	3.08E+06	727
**IL588**	1962	Cattle	Mice	Uganda	Uganda	6.88E+05	674
**ILC22**	1970	Cattle	Rat	Tanzania	Serengeti	3.58E+06	730
**ILC55**	1976	Dog	Mice	Kenya	Kabete	9.49E+05	454
**ILC66**	1976	Dog	Mice	Kenya	Kabete	3.72E+06	366
