# Supplementary material for: Variant antigen repertoires in Trypanosoma congolense populations and experimental infections can be profiled from deep sequence data using universal protein motifs
Source: Genome Res. 2018 Sep;28(9):1383–94. doi: 10.1101/gr.234146.118 (PMC6120623; doi:10.1101/gr.234146.118)
Supplement: Supplemental Material [file supp_gr.234146.118_Supplemental_Material.zip › Supplemental_Material/Supplemental_Table_S2.pdf]

Supplemental Table S2 Transcriptomics statistics and number of VSG transcripts recovered per sample for infection 2 (N=24).

Sample ID	Read pairs	Transcripts	Maximum FPKM	VSG transcripts	Maximum FPKM
**1**	3.19E+07	10695	3906.16	110	127.93
**2**	2.87E+07	9920	2505.62	63	270.35
**3**	2.32E+07	11073	2114.95	108	125.93
**4**	3.79E+07	9554	1798.98	52	183
**5**	2.24E+07	9501	2981.22	52	152
**6**	2.14E+07	8524	1355.24	43	138.16
**7**	2.82E+07	9619	4104.68	75	156.65
**8**	2.91E+07	6493	5621.37	36	155.84
**9**	3.50E+07	8374	1952.73	62	159.18
**10**	2.52E+07	6462	3119.37	46	191.9
**11**	2.77E+07	9768	4817.29	54	128.46
**12**	2.04E+07	6628	4026.51	31	131.78
**13**	3.23E+07	10694	2093.85	120	102.88
**14**	3.36E+07	7335	2729.18	79	431.31
**15**	4.00E+07	11437	2012.88	147	176.31
**16**	3.79E+07	8952	2368.46	68	173.21
**17**	3.24E+07	10811	1869.47	114	213.47
**18**	3.29E+07	11466	3003.4	94	222.41
**19**	4.13E+07	10456	2213.25	90	186.71
**20**	2.67E+07	10490	1754.01	114	150.43
**21**	3.49E+07	10243	1455.93	105	131.23
**22**	3.31E+07	8734	3346.27	118	634.24
**23**	3.16E+07	7200	1608.64	53	181.42
**24**	2.35E+07	9415	2696.13	66	316.45
