# Supplementary figures and images for: Variant antigen repertoires in Trypanosoma congolense populations and experimental infections can be profiled from deep sequence data using universal protein motifs
Source: Genome Res. 2018 Sep;28(9):1383–94. doi: 10.1101/gr.234146.118 (PMC6120623; doi:10.1101/gr.234146.118)

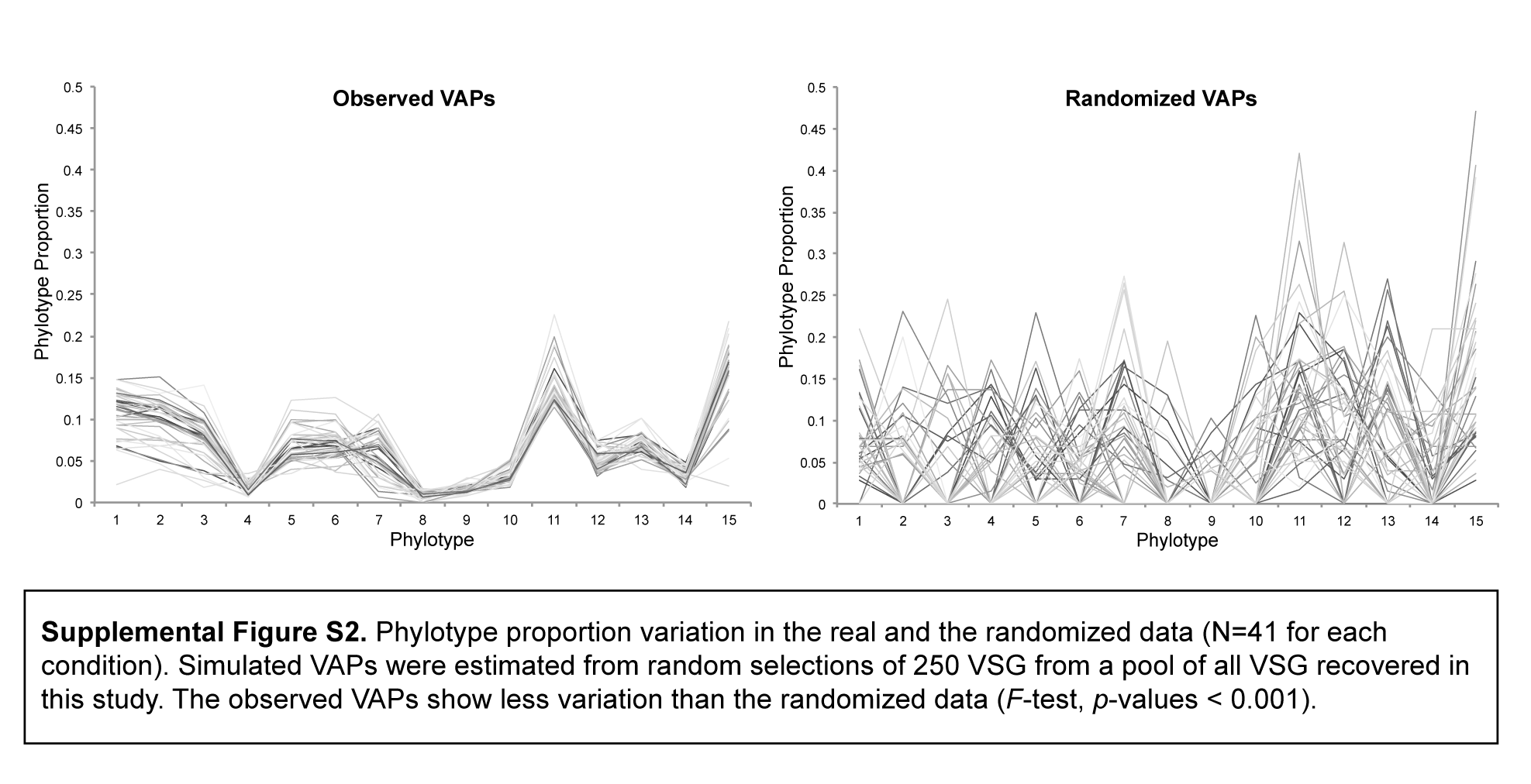

Supplement: Supplemental Material [file supp_gr.234146.118_Supplemental_Material.zip › Supplemental_Material/Supplemental_Fig_S2.tif]

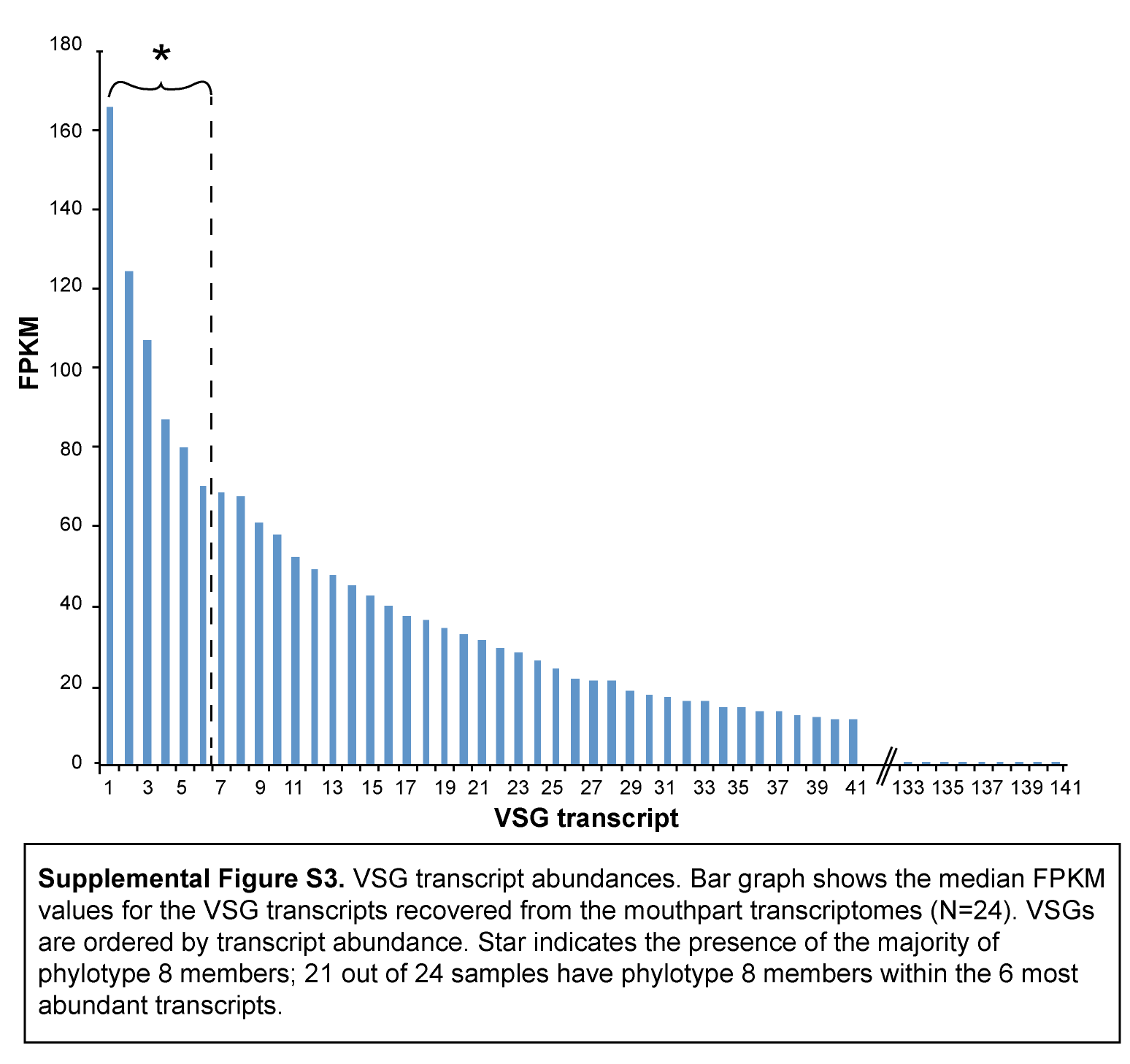

Supplement: Supplemental Material [file supp_gr.234146.118_Supplemental_Material.zip › Supplemental_Material/Supplemental_Fig_S3.tif]

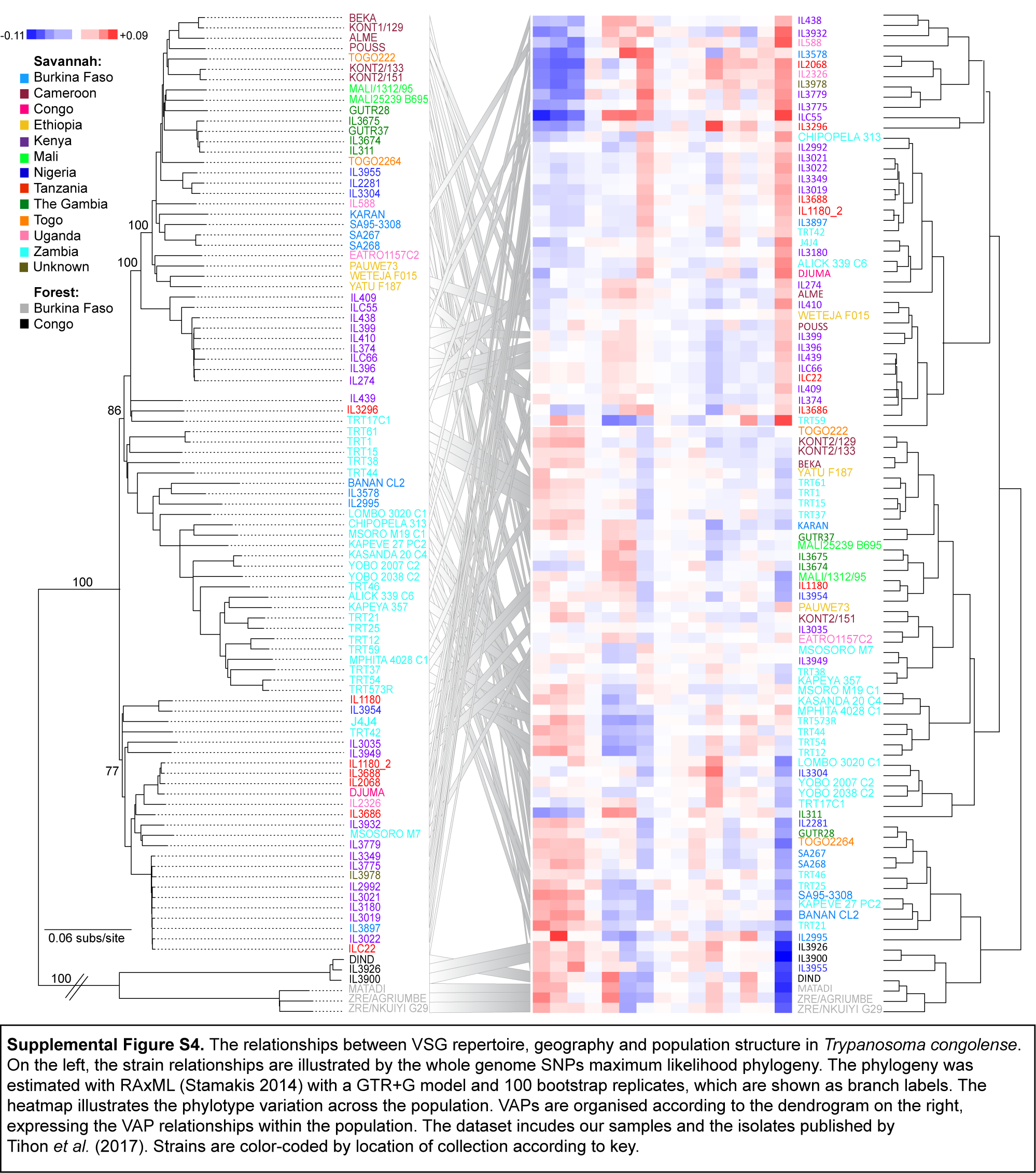

Supplement: Supplemental Material [file supp_gr.234146.118_Supplemental_Material.zip › Supplemental_Material/Supplemental_Fig_S4.tif]

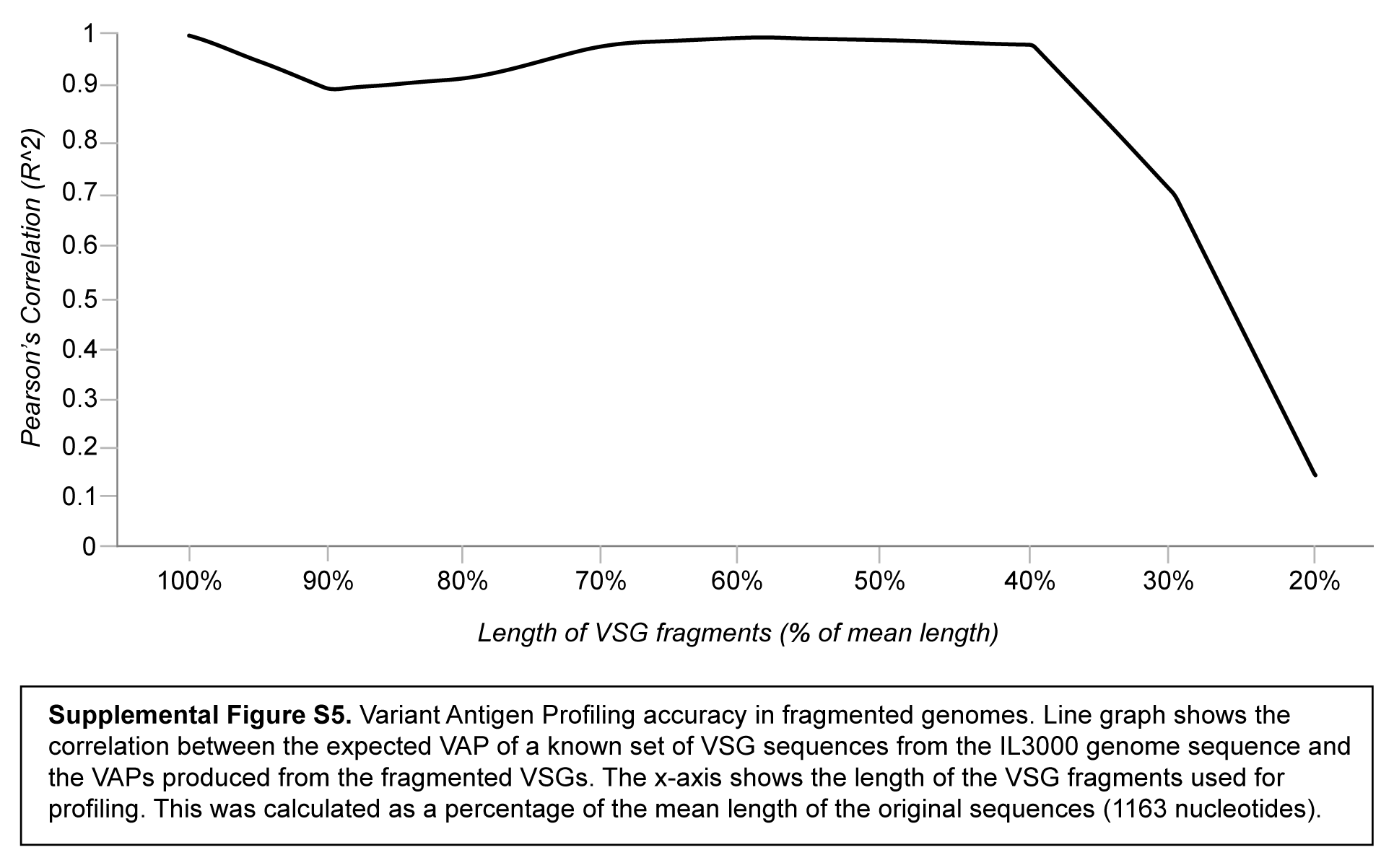

Supplement: Supplemental Material [file supp_gr.234146.118_Supplemental_Material.zip › Supplemental_Material/Supplemental_Fig_S5.tif]

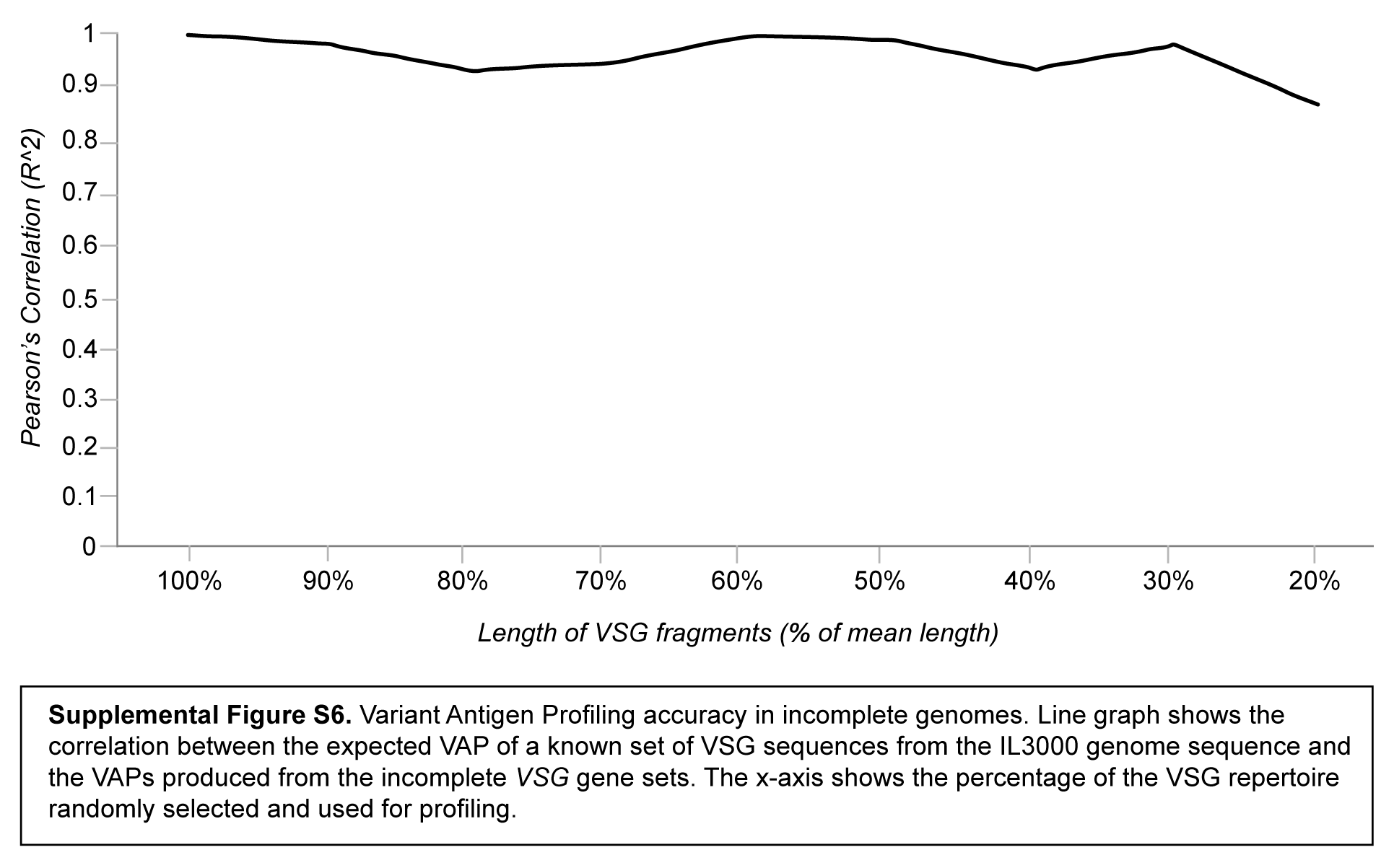

Supplement: Supplemental Material [file supp_gr.234146.118_Supplemental_Material.zip › Supplemental_Material/Supplemental_Fig_S6.tif]
